# Supplementary material for: Identification of photoperiod-induced specific miRNAs in the adrenal glands of Sunite sheep (Ovis aries)
Source: Front Vet Sci. 2022 Jul 22;9:888207. doi: 10.3389/fvets.2022.888207 (PMC9354845; doi:10.3389/fvets.2022.888207)
Supplement: Supplementary file 1 [file Data_Sheet_1.ZIP › Supplementary file/miRDeep2 file.zip!/miRDeep2 file/Know_Structure/SP42A2/oar-mir-26a.pdf]

miRBase precursor : oar-mir-26a
Total read count : 1591349
oar-miR-26a read count : 1591171
remaining reads : 178

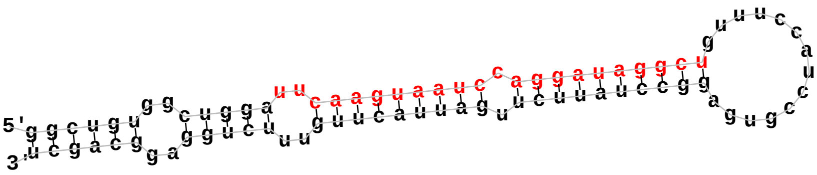

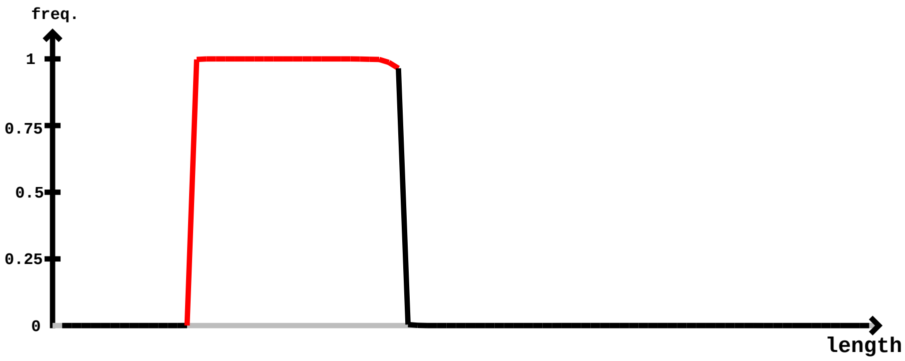


```
                oar-miR-26a
5'- ggcuguggcuggauucaaguaauccaggauaggcuguuuccauccgugaggccuauucuugauuacuuguuucuggaggcagcu   -3'    exp
    ((((((..(((((..(((((((((.((((((((((..............)))))))))).)))))))))..)))))..))))))    reads      mm         sample
    ............auucaaguaauccaggauagg...................................................    3          0          seq
    ............auucaaguaauccaggauaggc..................................................    14         0          seq
    ............Guucaaguaauccaggauaggc..................................................    2          1          seq
    ............auucaaguaauccaggauaggcu.................................................    257        0          seq
    ............Guucaaguaauccaggauaggcu.................................................    39         1          seq
    ............Guucaaguaauccaggauaggcug................................................    1          1          seq
    .............uucaaguaCuccaggau......................................................    2          1          seq
    .............uucaaguaauccaggaG......................................................    14         1          seq
    .............uucaaguaauccaggCu......................................................    1          1          seq
    .............uucaaAuaauccaggau......................................................    1          1          seq
    .............uucaaguaauccaggaA......................................................    14         1          seq
    .............uucaUguaauccaggau......................................................    2          1          seq
    .............uucaaguaauccaggau......................................................    812        0          seq
    .............uucaGguaauccaggau......................................................    6          1          seq
    .............uAcaaguaauccaggau......................................................    4          1          seq
    .............uucaaguaauccaggaua.....................................................    1353       0          seq
    .............uucaGguaauccaggaua.....................................................    11         1          seq
    .............uucaaguaauccaggauag....................................................    1133       0          seq
    .............uucaaguaauccaggauaC....................................................    2          1          seq
    .............uucaaguaauccaggauagg...................................................    17459      0          seq
    .............uucaaguaauccaggauaggc..................................................    34975      0          seq
    .............uucaaguaauccaggauaggcu.................................................    1525738    0          seq
    .............uucaaguaauccaggauaggcug................................................    4626       0          seq
    .............uucaaguaauccaggauaggcugu...............................................    1429       0          seq
    .............uucaaguaauccaggauaggcuguu..............................................    25         0          seq
    .............uucaaguaauccaggauaggcuguG..............................................    3          1          seq
    .............uucaaguaauccaggauaggcuguuu.............................................    3          0          seq
    ..............ucaaguaauccaggaua.....................................................    3          0          seq
    ..............ucaaguaauccaggauag....................................................    1          0          seq
    ..............ucaaguaauccaggauagg...................................................    24         0          seq
    ..............ucaaguaauccaggauaggc..................................................    75         0          seq
    ..............ucaaguaauccaggauaggcu.................................................    2944       0          seq
    ..............ucaaguaauccaggauaggcug................................................    26         0          seq
    ..............ucaaguaauccaggauaggcugu...............................................    10         0          seq
    ..............ucaaguaauccaggauaggcuguu..............................................    1          0          seq
```

oar-miR-26a

ggcuguggcuggauucaaguaauccaggauaggcuguuuccauccgugaggccuauucuugauuacuuguuucuggaggcagcu			
...............caaguaauccaggauagg...	5	0	seq
...............caaguaauccaggauaggc..	4	0	seq
...............caaguaauccaggauaggcu...	115	0	seq
................aaguaauccaggauagg...	1	0	seq
................aaguaauccaggauaggcu...	29	0	seq
.................aguaauccaggauaggcu...	2	0	seq
..................guaauccaggauaggcu...	1	0	seq
..................guaauccaggauaggcugu...	1	0	seq
....aggccuauucuugauuacuugu..............	2	0	seq
....aggccuauucuugauuacuuguu.............	10	0	seq
....aggccuauucuugauuacuuguuu............	3	0	seq
....ccuauucuugauuacuugu..............	5	0	seq
....ccuauucuugauuacuuguu.............	15	0	seq
....ccuauucuugauuacuuguuu............	63	0	seq
....ccuauucuugauuacuuguuuc...........	73	0	seq
....ccuauucuugauuacuuguuucu..........	3	0	seq
....cuauucuugauuacuuguuuc...........	1	0	seq
....cuauucuugauuacuuguuucu..........	2	0	seq
....auucuugauuacuuguu.............	1	0	seq
